# Anthropomorphic liver phantom with flow for multimodal image-guided liver therapy research and training

**DOI:** 10.1007/s11548-017-1669-3

**Published:** 2017-09-19

**Authors:** Anna Rethy, Jørn Ove Sæternes, Jostein Halgunset, Ronald Mårvik, Erlend F. Hofstad, Juan A. Sánchez-Margallo, Thomas Langø

**Affiliations:** 1 Department of Cancer Research and Molecular Medicine, Norwegian University of Science and Technology (NTNU), Trondheim, Norway; 2 Norwegian National Advisory Unit on Ultrasound and Image-Guided Therapy, St. Olavs Hospital, Trondheim University Hospital, Trondheim, Norway; 3 Department of Laboratory Medicine, Children’s and Women’s Health, NTNU, Trondheim, Norway; 4 Department of Gastrointestinal Surgery, St. Olavs Hospital, Trondheim, Norway; 5 Department of Medical Technology, SINTEF, 7465 Trondheim, Norway; 6 Department of Computer Systems and Telematics Engineering, University of Extremadura, Badajoz, Spain

**Keywords:** Liver phantom, Blood flow, Laparoscopic liver surgery, Ultrasound, Navigation

## Abstract

***Purpose*:**

The objective of this study was to develop a multimodal, permanent liver phantom displaying functional vasculature and common pathologies, for teaching, training and equipment development in laparoscopic ultrasound and navigation.

***Methods*:**

Molten wax was injected simultaneously into the portal and hepatic veins of a human liver. Upon solidification of the wax, the surrounding liver tissue was dissolved, leaving a cast of the vessels. A connection was established between the two vascular trees by manually manipulating the wax. The cast was placed, along with different multimodal tumor models, in a liver shaped mold, which was subsequently filled with a polymer. After curing, the wax was melted and flushed out of the model, thereby establishing a system of interconnected channels, replicating the major vasculature of the original liver. Thus, a liquid can be circulated through the model in a way that closely mimics the natural blood flow.

***Results*:**

Both the tumor models, i.e., the metastatic tumors, hepatocellular carcinoma and benign cyst, and the vessels inside the liver model, were clearly visualized by all the three imaging modalities: CT, MR and ultrasound. Doppler ultrasound images of the vessels proved the blood flow functionality of the phantom.

***Conclusion*:**

By a two-step casting procedure, we produced a multimodal liver phantom, with open vascular channels, and tumor models, that is the next best thing to practicing imaging and guidance procedures in animals or humans. The technique is in principle applicable to any organ of the body.

## Introduction

Laparoscopic resection of liver tumors is a demanding intervention for the surgeon. Laparoscopically non-visible tumors are difficult to localize, and it is challenging to find the optimal resection border to avoid vessels located close to the tumor. Intraoperative ultrasound [1], in combination with a navigation system [2], can provide guidance by showing the position of surgical instruments in relation to preoperative images (MR/CT). Ultrasound (US) provides real-time imaging in the operating room, including functional imaging such as blood flow (Doppler). However, the image quality of intraoperative ultrasound is low compared to CT or MR, i.e., images can be difficult to interpret and tumors as well as vessels difficult to delineate. Additionally, navigation systems that incorporate all these different imaging modalities have a steep learning curve. There is thus a demand for a training device. A life-like phantom, with correct anatomic detail, may fulfill this need. During laparoscopic liver surgery, the blood vessels in the liver are differentiated from other structures using Doppler ultrasound. To mimic this feature, the phantom must comprise functionality for flow through an anatomically realistic blood vessel system. Moreover, the phantom should contain models for training localization of pathological lesions.

There are liver phantoms available on the market, or described in the scientific literature, with tumor models, blood vessels structures [3, 4] and real appearance [4], but none of them provide blood flow functionality.

The objective of this project was to create a phantom that mimics real human organs (primarily the liver) with respect to vasculature and common pathologies, producing realistic images in commonly used medical imaging modalities such as CT, MR and ultrasound. The phantom should be suitable for teaching, training and equipment development purposes in the field of laparoscopic ultrasound, especially the combination of preoperative imaging (CT, MR) with real-time intraoperative ultrasound using tracking technology.

Below, we describe how the phantom was created. We present its multimodal usefulness by providing CT, MR and ultrasound images. Further, an experimental setup is presented to demonstrate its functional aspects with blood flow and Doppler imaging. Finally, we show the phantom in an experimental intraoperative surgical navigation setting.

## Background

### Liver phantoms

Regarding the commercially available liver phantoms, the Triple Modality 3D Abdominal Phantom (CIRS Inc., Norfolk, VA, USA) [5] provides a training model for ultrasound-guided needle insertion and navigation technology. This represents a slice of the abdominal cavity that includes parts of the liver, tumor models and blood vessel structures. The CIRS phantom allows for multimodal medical imaging (US, CT and MR). Another commercial phantom, representing the upper abdominal organs is the IOUSFAN${\circledR }$ (Kyoto Kagaku Co., Ltd, Kyoto, Japan) [6]. This is designed as a practice and demonstration tool for abdominal intraoperative and laparoscopic ultrasound. It has realistic appearance and provides tumor models and blood vessels structures.

Most of the liver phantoms developed for research purposes have been intended for CT imaging applications [7–13]. Fewer phantoms have been developed for MRI [14, 15] and ultrasound [16–18]. Some of the latter include vascular structures [15–17] and have a realistic appearance [16, 17]. Multimodal liver phantoms are still scarce [3, 4, 19, 20]. To the best of our knowledge, the present study is the first describing a multimodal liver phantom based on real human liver anatomy, which includes tumor models, vascular structures and blood flow functionality.

### Phantom materials

In the literature, a multitude of techniques and tissue-mimicking materials have been proposed to prepare phantoms. The bulk matrix materials for soft tissue mimicry have been based on aqueous suspensions, agarose, gelatin, magnesium-silicate, oil gel, polyacrylamide gel, polyurethane resin, polyvinyl alcohol (PVA), polyester resin, epoxy resin, polysaccharide gels TX-150 and TX-151, polyacrylamide and room-temperature-vulcanizing (RTV) silicone [21–23]. Agarose- and gelatin-based gels (hydrogels) are the most widely used alternatives for soft tissue modeling described in the literature [21, 22]. They are well-characterized and can be prepared with a range of acoustic properties [21], but they usually have a limited life span [23]. Oil gel-based phantoms have a propylene glycol or ethylene glycol base. The main advantages of propylene glycol are its resistance to bacterial colonization and its linear increase of speed of sound and attenuation with the proportion of propylene glycol. They also contain a gelatinizer and polymethyl methacrylate microspheres [24]. Ethylene glycol-based oil gels tend to be too dense, with low attenuation. Polyurethane, polyester and epoxy resins have good mechanical characteristics for mimicking soft tissue [24]. Standardization of the polyurethane gel-based phantoms production, however, is problematic due to complex molecular design of the gels. PVA-based models (cryogels) are of low cost and indefinite longevity, but the production process is highly demanding [25, 26]. Polysaccharide gels are inexpensive, conveniently moldable and temporally stable [27], but it may be difficult to avoid air bubbles, which constitute an issue for ultrasound imaging. Polyacrylamide gel-based tissue substitutes are made by polymerization of highly toxic acrylamide monomer [28], therefore, rendering special precautions mandatory. RTV silicone is easy to work with [29] and provides a soft rubbery texture similar to that of tissue. The major shortcomings of this material are high cost and extended hardening time.

The four most common materials used for ultrasound scattering particles are lipid microparticles, polymer microparticles, metal oxide powders and quartz glass microspheres. Lipid microparticles of 10–500 nm are biologically analogous to bilipid membranes of cells and organelles, which are believed to cause scattering in tissue. Commercially available lipid-based scatterers are milk [30, 31], fat/oil/lipid [32] and Intralipid/Nultralipid [33–35]. Polymer microspheres of 50–100 nm are produced in well-controlled sizes, which means that repeatability and predictions of spectra are good [23]. Wide availability of TiO$_{2}$ powder, 20–70 nm, makes titanium dioxide one of the most commonly used scatterers. However, it settles when not stirred, which is a problem when fabricating aqueous suspensions. Therefore, TiO$_{2}$ powders should preferably be used with gelatin- or agarose-based, RTV and resin phantoms. The use of quartz glass microspheres (250 nm) is less well established [23].

### Blood vessels and flow concept

All organs in the human body have a vascular blood supply. There is at least one arterial blood vessel leading blood into the organ, and there is at least another taking blood away from it. The connection between the arterial and venous vascular trees is through a capillary network, on a microscopic level.

## Materials and methods

The presented anthropomorphic liver phantom consists of four main model components: the bulk of tissue corresponding to the liver parenchyma, blood vessels, tumors and the gallbladder. Connecting vessels are visible on the surface, and internal vessels are inside the liver model.

The vasculature is patent, and it consists of two interconnected vascular trees. One allows for fluid infusion into the phantom, the other provides the port of fluid exit. The connection between these two vascular trees is a physical end-to-end connection of all corresponding branches. Flow through the phantom is created by directly connecting the portal vein end and the vena cava end to a pump, by the intermediary of flexible rubber tubes. Fluid is pumped into the portal vein and flows out through the hepatic veins that drain into the supra-hepatic portion of the inferior vena cava, from here on referred to as vena cava for simplicity.

### Liver mold

**Fig. 1:**
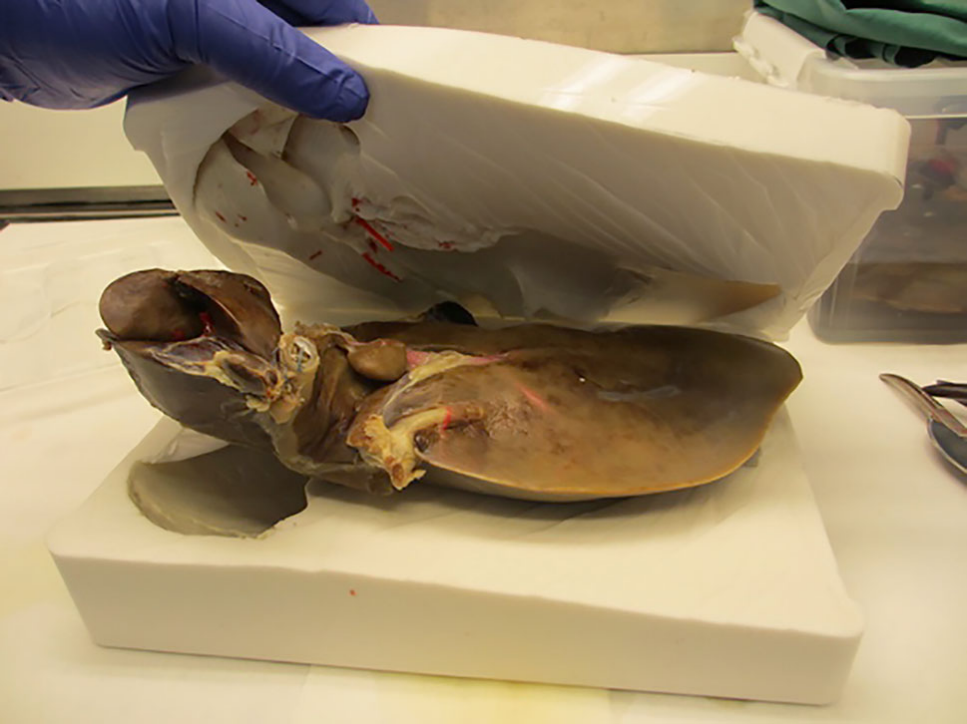
Development of the mold from an anatomic liver specimen

Due to its accurately preserved shape, a cadaveric human liver specimen was used as the starting point (Fig. 1). After careful preparation and dissection, a three-part silicone mold was made from the liver using Elasturan 4503 (BASF Polyurethanes GmbH, Lemförde, Germany).

### Parenchyma

Polyurethane was chosen to develop the parenchyma due to its durability and liver-like consistency. To this end, Elasturan 6005/264 (BASF Polyurethanes GmbH) -100 pbw (parts by weight), and ISO 136/131 -20 pbw- with 0.6% Sephadex G2580 (Sigma-Aldrich, San Luis, MO, USA) bw (by weight) were used as a volume spreader. Components were mixed by hand, and subsequently vacuumed at 15 mBar pressure to eliminate air bubbles.

### Blood vessels

#### Internal liver vessels

The internal vascular components were produced using corrosion casting. This is an established method of creating an exact replica of the 3D vascular structure of organs and tissues [36, 37]. The blood volume is replaced by a fluid material, which becomes solid after instillation. Thereafter, the surrounding tissue is removed by alkali to reveal the vessel cast. For injection into the blood vessels we selected *Tissue Tek III* ${\circledR }$ *– Paraffin* (Sakura Finetek Europe B.V., Alphen aan den Rijn, The Netherlands), due to its low melting point of 54–57 ${^{\circ }}$C, malleability with moderate heat and relative break resistance due to added polymer.

**Fig. 2:**
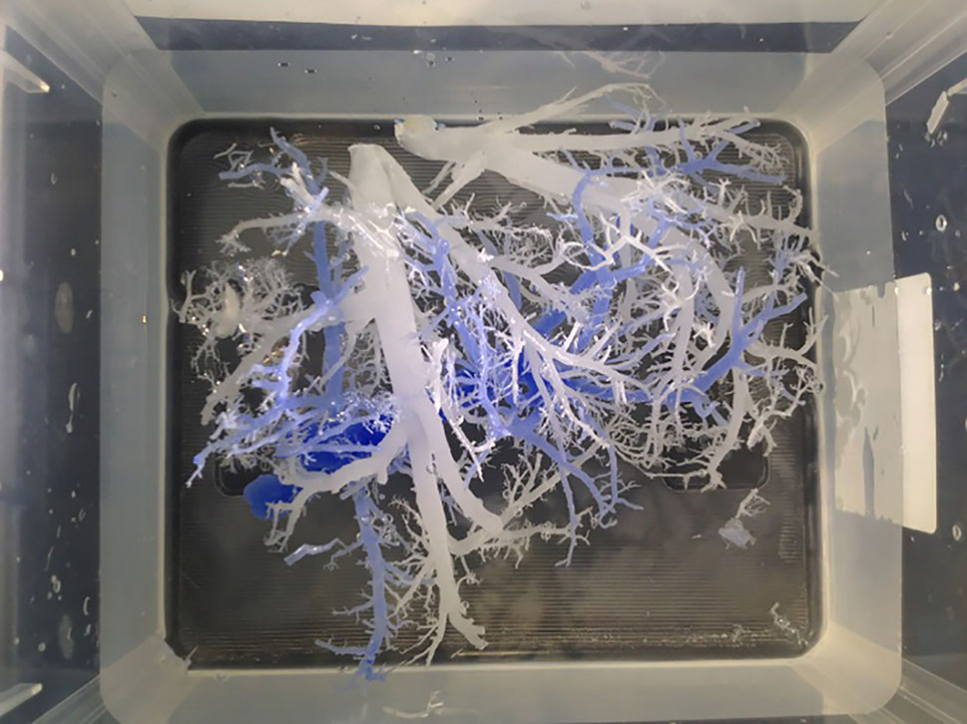
Paraffin casts of the two parts of the liver vascular tree

A donated fresh cadaver liver was injected with the hot liquid paraffin simultaneously in the portal vein and in the supra-hepatic portion of the inferior vena cava, with a blue dye added to the paraffin injected into the vena cava. After hardening of the paraffin, the liver was placed in a 30% potassium hydroxide (KOH) solution. The tissue dissolved within 48–72 h, leaving behind only the two separate paraffin casts (Fig. 2). The branches of the casts were preserved down to a diameter of 2–3 mm, whereas smaller branches were discarded.

#### External liver vessels

A part of the portal vein and parts of the vena cava were replicated as hollow tubular structures made of the same mixture as the parenchyma. We used molds of two different sizes:

- *Large* A three-part mold was constructed from:
  - Poly(methyl methacrylate) (PMMA) tubing with an inside diameter of 25.7 mm, and a length of 14 cm, divided lengthwise in half.
  - PMMA tubing with an outside diameter of 20 mm.
- *Small* A three-part mold was constructed from:
  - PMMA tubing with an inside diameter of 15.7 mm, and a length of 14 cm, divided lengthwise in half.
  - PMMA tubing with an outside diameter of 10 mm.

For both sizes, the smaller PMMA tube was placed inside the larger one and the artificial vessel was cast between the two tubes using the same mixture as the parenchyma.

The vena cava model was assembled by joining one *large* and three *small* vessels, the smaller ones representing the hepatic veins, that drain into the larger one representing the vena cava. The four parts were glued together with the same material. The larger portion of the vessel extends 3 cm above the surface of the liver superiorly and continues the inferior posterior portion of the liver where it terminates.

The portal vein model was assembled from three parts, one*large *and two *small* vessels. It enters the liver inferiorly, branching into right and left branches after it enters the liver. A 3-cm-long portion of the portal vein on the outside of the liver and the two branches serve for correct placement of the two branches on the inside.

The two vascular components, internal and external, all together four parts, were assembled together in the liver mold to produce one continuous hollow structure in the end.

### Tumors

Three different types of tumor models were created and integrated in the phantom: metastatic lesions, hepatocellular carcinoma and benign cysts. The basic material for the tumors was the same as for the parenchyma with the addition of 5% calcium carbonate bw (0.5 g calcium carbonate powder/10 g of polymer). Cyst walls were made using parenchyma material only.

#### Metastasis

Metastases were created as spherical models corresponding in shape and size to lesions commonly met in clinical practice. Two different sizes, with diameter of 1.1 and 1.6 cm, were created using two-part silicone molds.

#### Hepatocellular carcinoma

Since hepatocellular carcinomas have highly irregular shapes, tumors were created by pouring the mixture directly onto a branching of vessel casts in an irregular manner.

#### Benign cysts

Models of benign cysts were created using the same mold as the one used for the metastases. A balloon structure was created by coating the inside surface of the spherical mold with parenchyma material. After curing, the balloons were filled with water and then sealed.

### Gallbladder

A two-part silicone mold was produced using an anatomic specimen as basis, modeling the body of the gallbladder, the cystic duct and the common bile duct. The right and left bile ducts run approximately in parallel to the right and left branches of the portal vein. Parenchyma material was used to create the gallbladder.

### Phantom assembly

**Fig. 3:**
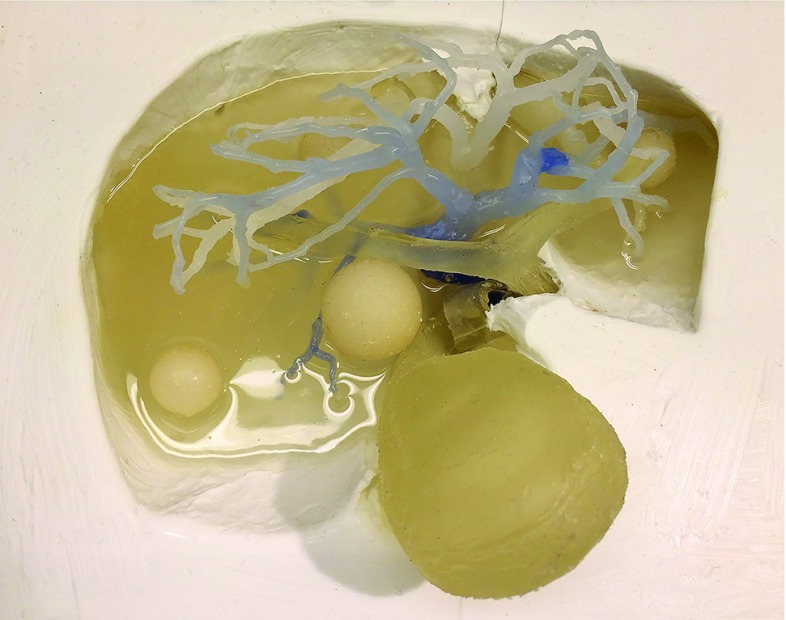
Layer 2 casting picture displays: the two vascular tree casts of the portal and the cava system with corresponding ends connected the gallbladder with the bile ducts and two tumors of different sizes

The two tubular vessel models were secured in place in the bottom two portions of the liver mold. The gallbladder was secured in place. The two vascular tree casts of the portal and the cava system, respectively, were inserted in the open end of the corresponding vessel model and fixed. The free ends of the two cast trees were joined together manually by softening/melting the paraffin. Care was taken to keep the corresponding bile ducts alongside the branches of the portal vein. The parenchyma substitute was poured into the mold in three layers, and tumor models were added at vessel junctions in layers 2 and 3 (Fig. 3). Before addition of the tumors, each layer was vacuumed for 7 min at 15 mBar pressure. Complete curing was allowed between each layer. Layer 3 was added after the 3rd, and top part of the mold was secured in place. The final phantom cured for 5–7 days.

**Fig. 4:**
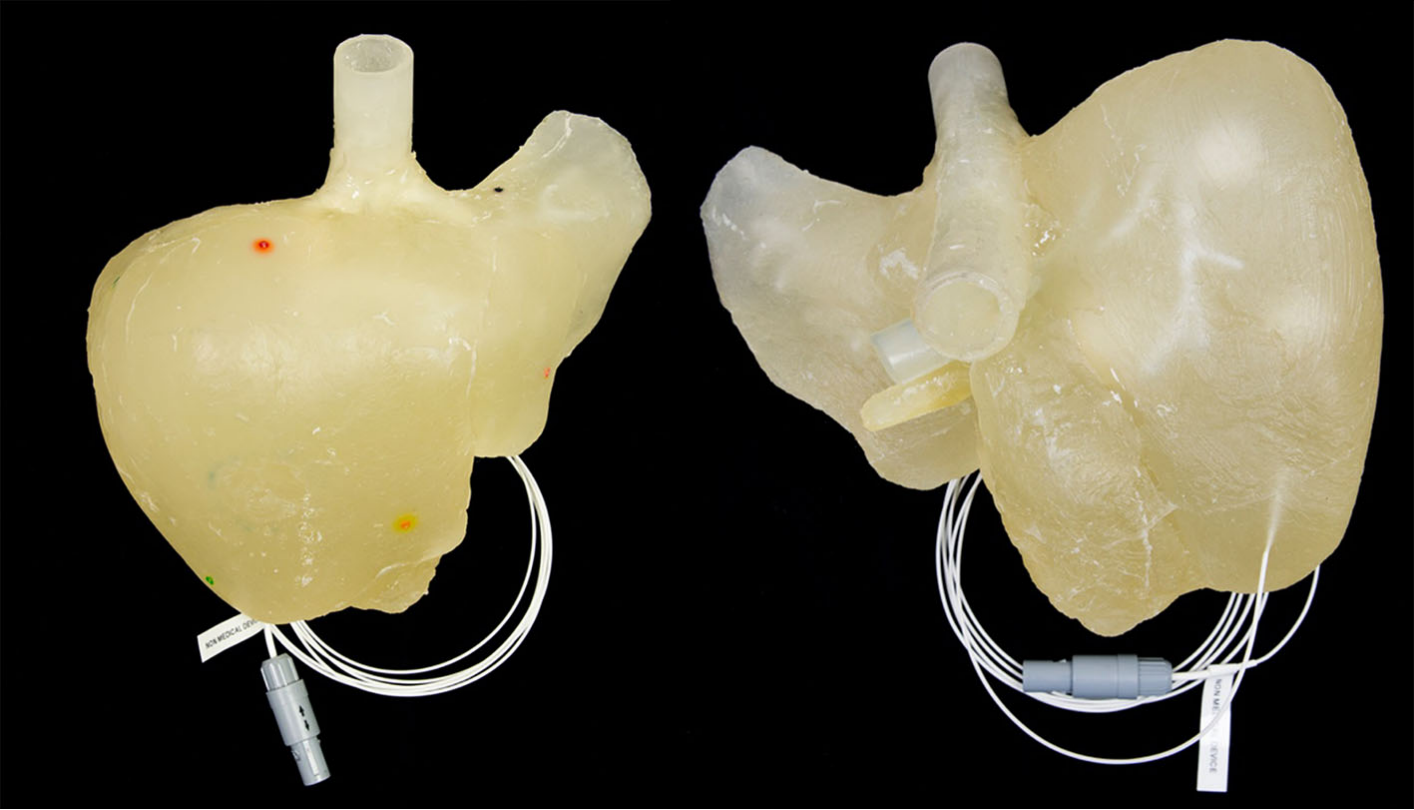
ANJO liver phantom showing the colored depressions (left picture), 1.5 mm deep and wide, which serve as positional markers for navigation. The EM tracking sensor incorporated inside the parenchyma 5 mm below the surface is shown in the right picture

Once cured, the phantom was placed in an 80${^{\circ }}$C water bath for 1/2 h to allow for the paraffin to begin melting. The phantom vascular tree was flushed with running water of the same temperature, first through the vena cava, allowing the paraffin to flow out through the portal vein. The same process was repeated starting with the portal vein, and continued until only clean water was seen to emerge from the vena cava. On the finished phantom, six depressions were carved on the anterior surface corresponding to the six front lobes of the liver. The depressions, 1.5 mm deep and wide, serve as positional markers for navigation. They are clearly visible by CT and MR. The depressions were stained, each with a different color, for accurate visual identification (Fig. 4). An electromagnetic (EM) tracking sensor was incorporated inside the parenchyma 5 mm below the surface for use with the navigation system (Fig. 4). The navigation platform, CustusX [38], is capable of importing preoperative data like CT, reconstructing these into 3D, segmenting structures from the CT data, reading in real time the tracked ultrasound probe, multimodal visualization techniques, registration of images to the phantom (or patient) and also image-to-image matching techniques. Navigation is further explained, and example images are shown in the Results section.

## Results

**Fig. 5:**
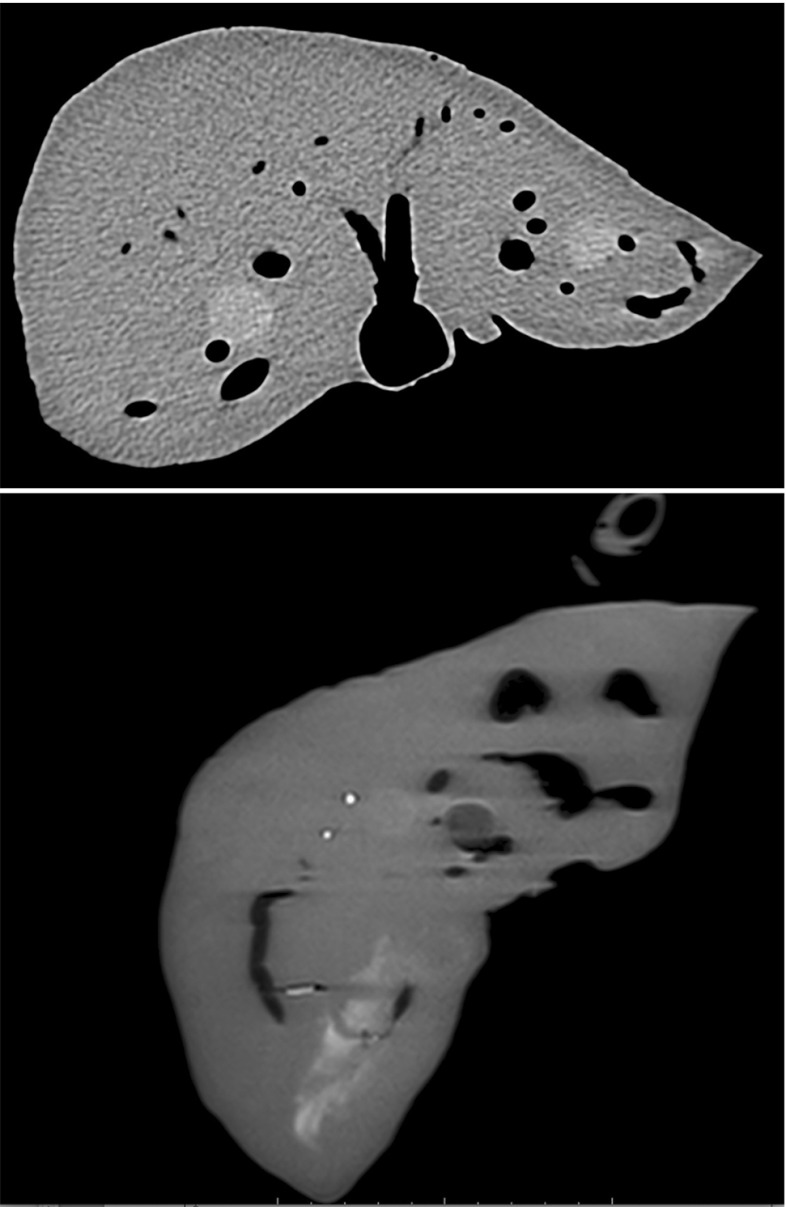
CT (top) and MR (bottom) images of the phantom showing cross section of vessels and metastatic tumors. Hepatocellular carcinoma visible on MR picture. Bright marks on MR image are due to remnants of wax

**Fig. 6:**
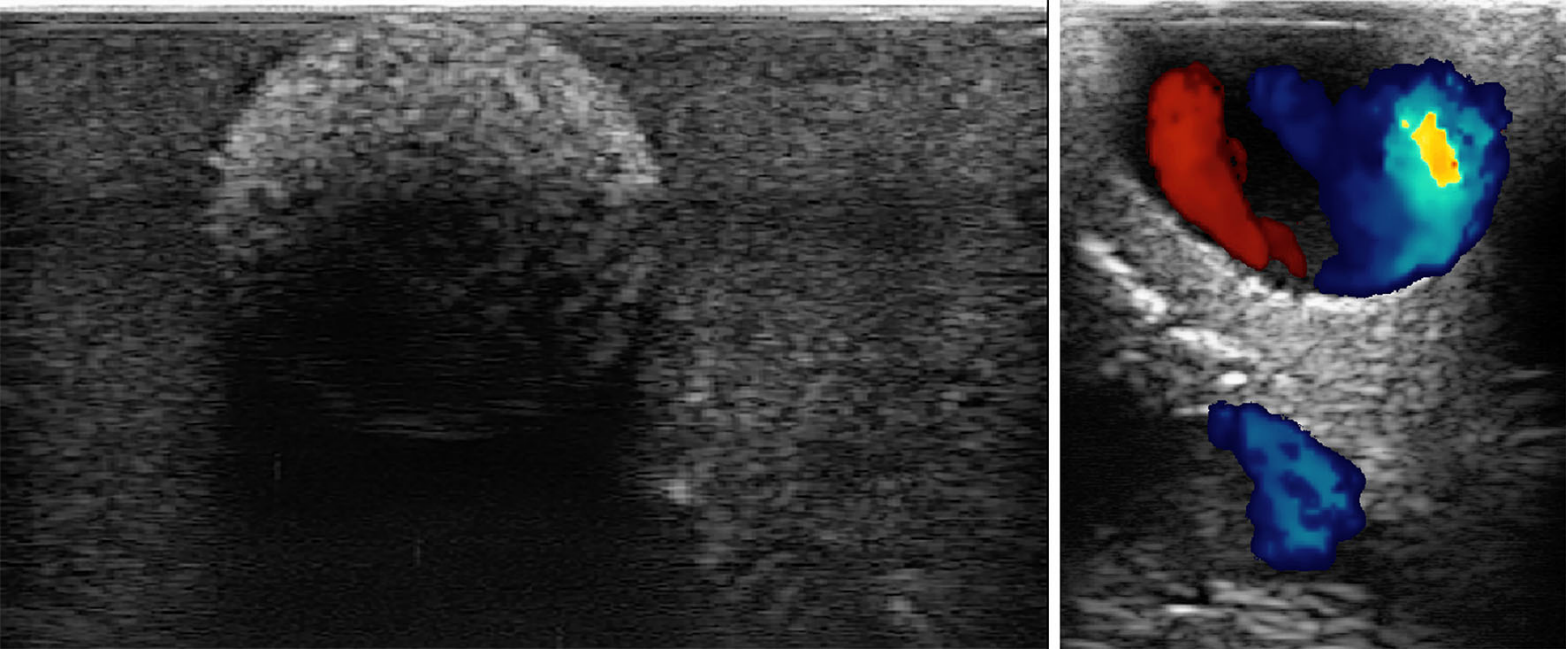
Ultrasound images of the phantom. Left: metastatic tumor model. Right: color Doppler ultrasound of vessels with circulating fluid

**Fig. 7:**
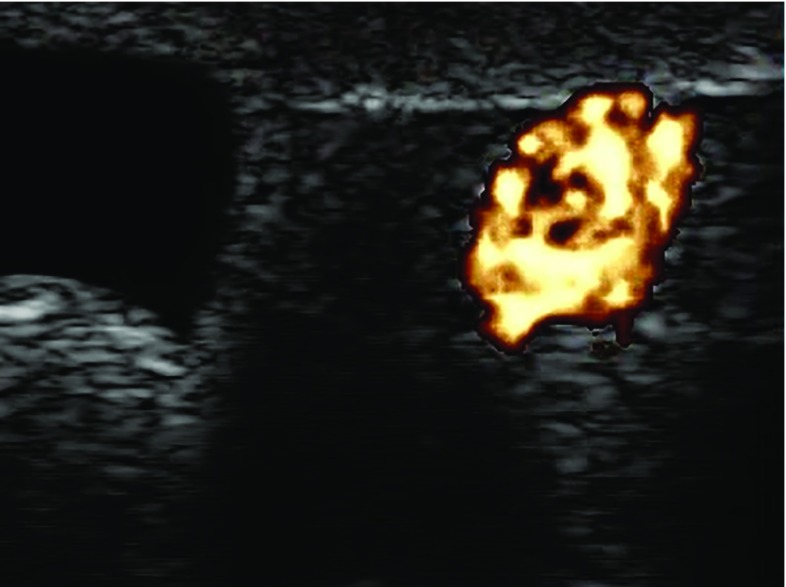
Ultrasound image of a benign cyst model (left) and vessel (right) with flow. Hepatocellular carcinoma tumor model surrounds both hollow structures

**Fig. 8:**
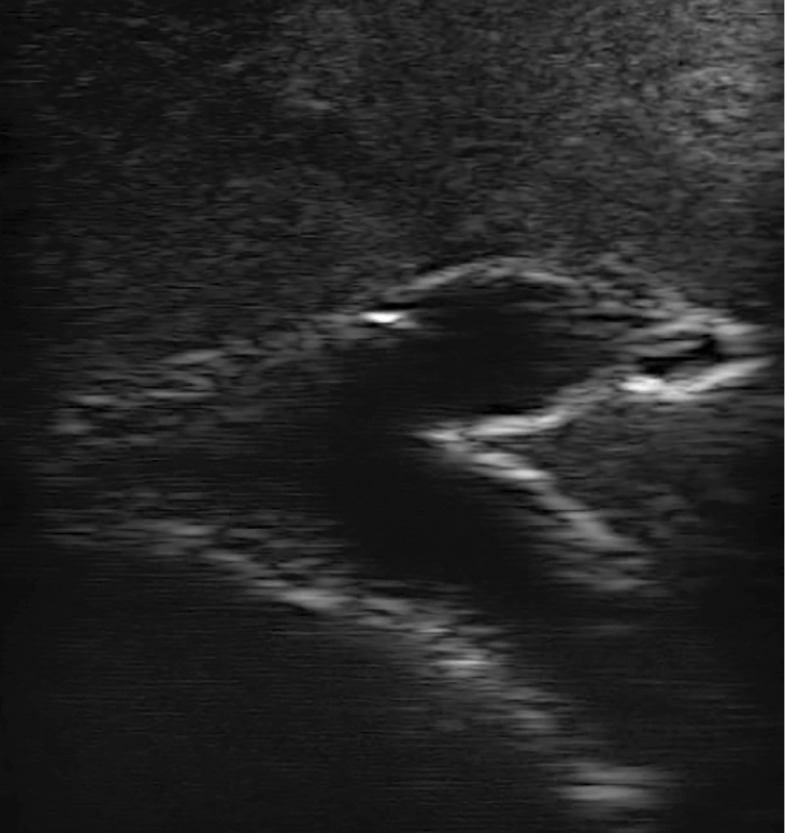
Ultrasound showing a vessel bifurcation with infiltrating hepatocellular carcinoma tissue

The multimodality of the phantom was tested by imaging with CT, MR and ultrasound. The image quality was assessed by the ability to discriminate between the different structures in the images. Figures 5, 6, 7 and 8 show sample CT, MR and ultrasound images of the liver phantom depicted in Fig. 4. Both the tumors and the vessels inside the liver model were clearly visualized by all the three imaging modalities.

### CT

CT images of the phantom were acquired using Dyna CT (SIEMENS, Berlin, Germany), Siemens Artis Zeego and a liver scan protocol with 629 slices of 512 $\times$ 512 pixels. The image resolution was 0.422 $\times$ 0.422 mm, and the slice thickness was 0.6 mm with 0.3 mm between slices.

### MR

MR images were acquired using a 1.5 Tesla scanner (SIEMENS) with a T1-weighted Abdomen Liver scanning protocol. Thus, 160 images were recorded, each with 512 $\times$ 512 pixels. The image resolution was 0.5 $\times$ 0.5 mm, and the slice distance was 1.0 mm.

### Ultrasound

**Fig. 9:**
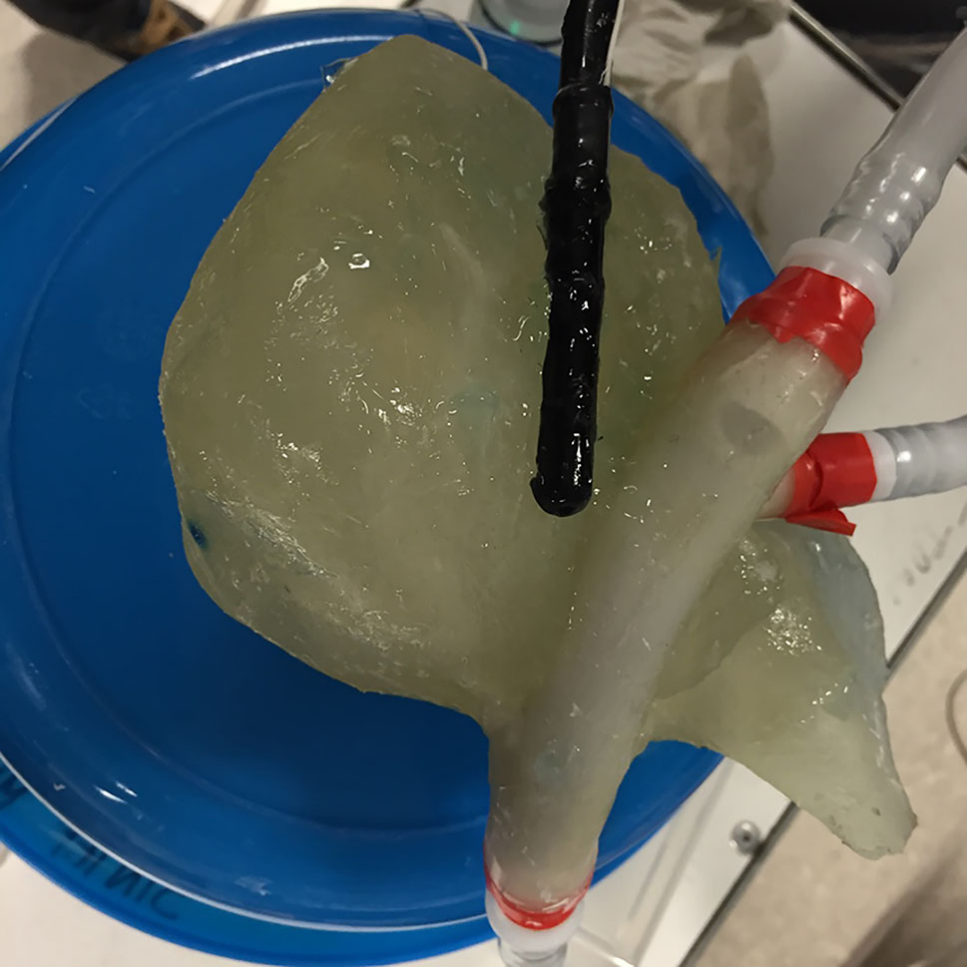
The experimental setup with the phantom connected to a flow system. The sensor is attached using a medical grade elastic shrinking tube (Olympus, Germany)

Ultrasound images of the phantom were acquired using the Ultrasonix MDP scanner (Analogic Ultrasound, Canada) with a laparoscopic probe (4-way flexible linear array probe, Vermon, Tours, France) (Fig. 9).

According to the difference in time of the received signal, sound speed of Elasturan was determined to be 1425 m/s (reference value of soft tissue is 1480 m/s). Attenuation at 5 MHz was 2 dB/cm/MHz, whereas at 2 MHz it was 1 dB/cm/MHz. Attenuation was not affected by the concentration of Sephadex. The concentration of calcium carbonate added to the parenchyma mixture to create the tumors caused realistic looking contrast between tumor and parenchyma with minimal shadowing. The benign cyst models appear anechoic in the ultrasound images, while metastatic lesions appear hyperechoic.

### Doppler

Figure 6 shows the Color Doppler ultrasound of internal vessels of the phantom with flow, and Fig. 7 shows the Power Doppler ultrasound of a vessel structure next to a cyst model. Larger and smaller vessels were identified. The outflow through the vena cava was regulated by using a clamp in order to provide enough pressure in the portal vein to ensure blood flow through the smaller vessels in the liver model.

### Ultrasound navigation

The phantom was evaluated using the module for laparoscopic surgery in the research navigation platform CustusX (SINTEF, Trondheim, Norway, http://www.custusx.org) [38].

**Fig. 10:**
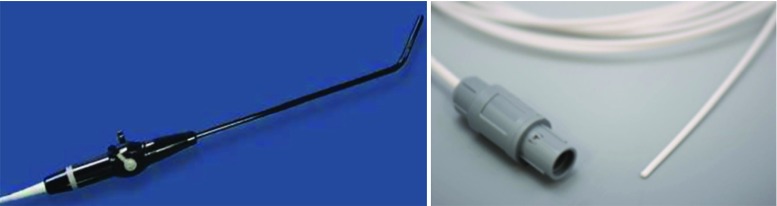
Laparoscopic ultrasound probe (left) and EM tracking sensor (right)

CT images of the phantom were imported into the navigation system and visualized in both 2D cross-sectional slices and a 3D model. The digital ultrasound video stream was enabled by a connection to an Ultrasonix MDP scanner. A model of the ultrasound probe and the real-time ultrasound images were visualized using CustusX. The ultrasound image was tracked with an electromagnetic tracking (EMT) sensor (Aurora, NDI, Ontario, Canada) mounted at the tip of the probe (Fig. 10). The position of the ultrasound image in relation to the sensor was calculated through a calibration procedure [39]. The ultrasound probe and images were aligned in the corresponding position and orientation to the CT images through a landmark-based registration procedure. Three markers on the liver phantom surface (described in the phantom assembly section) were pinpointed both in the CT image and in the tracking system by using an EMT pointer. The registration matrix was obtained by minimizing the total distance between the corresponding points.

**Fig. 11:**
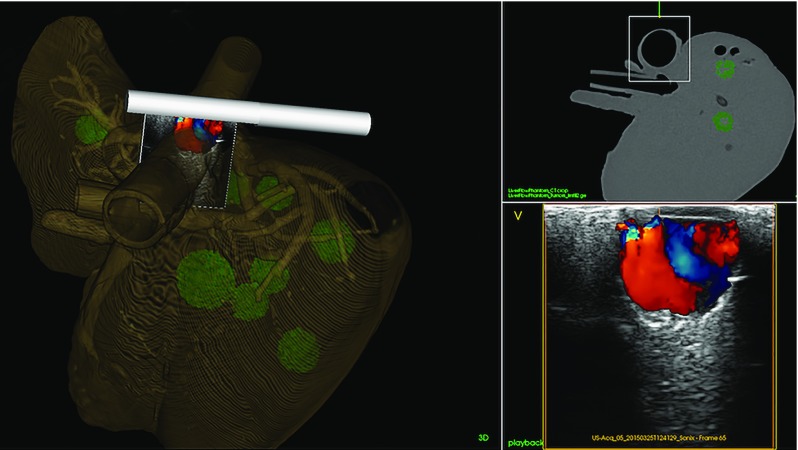
The navigation scene from CustusX. Left: 3D model of phantom (CT) overlaid with live ultrasound. Top right: the position of the ultrasound sector in relation to the CT volume. Bottom right: live ultrasound with color coded Doppler signal

Figure 11 shows the navigation scene from the CustusX navigation research platform. After calibration, the real-time ultrasound and preoperative CT images were visualized together in the same images using the navigation pane. This included a 3D scene with the ultrasound overlaid onto the CT image. This produced a 2D slice of the CT image projected to the spatial positions of the ultrasound image sector, thus visualizing the part of the CT volume corresponding to the real-time tracked ultrasound image.

## Discussion

We succeeded in establishing a method for producing a lasting artificial liver phantom based on real organ anatomy, and including flow functionality. We were also able to demonstrate multimodal imaging capabilities of the phantom. The main motivation for this project was the lack of suitable organ phantoms for training, with realistic mimicry of vasculature and blood flow, compatible with modern diagnostic imaging tools.

The gallbladder model was not realistically visualized either on CT or MR. On ultrasound, the contrast between it and the liver parenchyma did not correspond to a real situation. The purpose of the gallbladder at this stage in the phantom development process was to aid in development of the assembly technique. Plans exist of producing a modified version the gallbladder mold, similarly to the one mentioned above for the cyst, using the same material, resulting in a hollow structure. The hollow gallbladder along with its ducts would be filled with water and sealed, thereafter, incorporated into the phantom as described above.

A summary of the main features of the different liver phantoms developed for research purposes is presented in Table 1. The only commercially available alternative with a functionality close to the presented anthropomorphic liver phantom is the CIRS phantom [5]. However, its blood vessels do not allow flow, and the phantom does not have realistic appearance in the images. Needle track marks remain visible, and hence, the phantom has a limited life span. This may be considered a technological tool for system calibration rather than a clinical tool for development of navigation skills with ultrasound. The main limitations of the IOUSFAN$\circledR$ phantom [6] are that it is not multimodal, the internal anatomy is not truly realistic, blood flow cannot be simulated, and it is costly. Regarding the research phantoms, only the one created by Widmann et al. [13] has a realistic shape for the tumor models and provides blood vessel structures. They designed an anthropomorphic non-rigid phantom for evaluation of CT imaging systems, including vascular structures such as portal and hepatic veins. Liver phantoms with multimodal imaging capabilities are scarce [3, 4, 19, 20]. Among them, only the phantom presented by Chmarra et al. [3] allows for the combination of ultrasound, CT and MR imaging modalities. The authors presented a cost-effective and reusable phantom for development of image-guided therapeutic interventions and diagnostic imaging techniques and systems, consisting of three types of mimicked soft tissues such as liver parenchyma, tumors and blood vessels. Shevchenko et al. [4] also designed a multimodal liver phantom with vascular structures, and a more realistic appearance. They proposed a phantom for testing navigation systems and liver resection training, providing vascular trees and tumor models. However, none of the liver phantoms mentioned above, with vascular structures, can provide blood flow functionality.

For automation, combinations of 3D printing and casting may be used to achieve similar results. Multiple 3D printers are currently available, and three types have been used in medicine: (a) extrusion, (b) powder bed and (c) light polymerized printers [40]. Each of these printers uses a different fabrication material. O’Brien et al. [40] presented a critical review of 3D printing for the development of medical training models, with a focus on transplantation medicine. They identified three areas where 3D printing is currently used: (a) printing patient specific models for preoperative planning, (b) printing educational models for use by trainees to practice before patient care and (c) 3D printed molds for soft tissue casting. Our phantom addresses the last two of these uses.

**Fig. 12:**
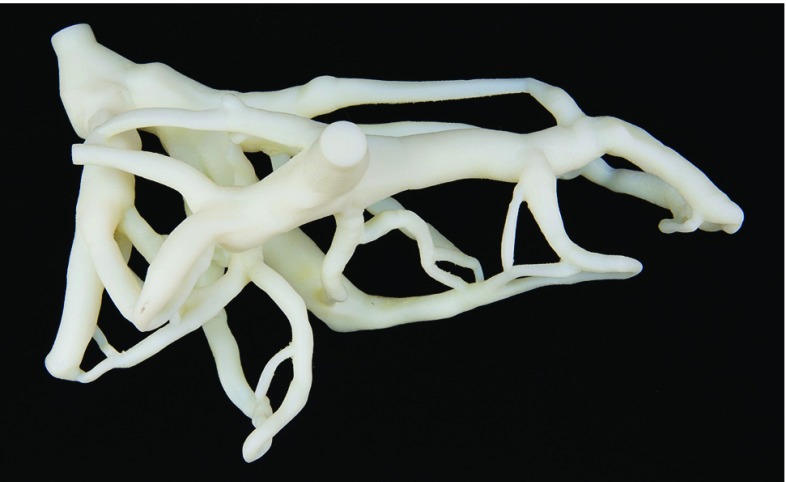
3D printed liver vascular system using ABS

Both the mold and the vascular structure can be 3D printed to eliminate the need for a human liver. In Fig. 12 we show an example of the 3D printed model of a hepatic vascular structure. This 3D printed vascular system could be removed, just like the wax, creating the negative space, and ultimately achieving the same result as with the corrosion cast model. Others have also used 3D printing for achieving realistic models of organs for surgical assistance. Thus, Kusaka et al. [41] developed a kidney graft and pelvic cavity replica. However, they did not address the multimodal imaging aspect nor the flow functionality. The main goal of this study was to produce a phantom that fulfilled several requirements in one phantom:

- Durable
- Flow (functional)
- Anatomically realistic, normal and pathological structures
- Multimodal

The use and application of the multimodal liver phantoms are numerous:

1. Training of general practitioners in performing simple ultrasound diagnostics on liver, for example, with gallbladder disease.
2. Training of surgeons (gastric, urologic, endocrine) performing laparoscopy or other minimally invasive surgical procedures, planning and practicing virtual resections, practicing ultrasound-guided needle insertions, radio frequency (RF) ablations, biopsies or demonstration of procedures.
3. Practice and education in several groups: radiologists, medical students, medical imaging engineers, imaging system developers (ultrasound, CT, MR), surgical navigation system developers and researchers within the field of medical technology.
4. Comparison of performance between imaging systems.
5. Establishment of appropriate training for translation of new imaging technologies in clinical practice
6. Training of novices learning ultrasound tissue characterization and ultrasound image interpretation in relation to anatomy.
7. Assessment of ultrasound acquisition and 3D reconstruction based on knowledge about sizes and shapes of the phantom constituents both from the production and from CT and/or MR images.

The trend of minimally invasive surgery is not limited to the liver. The methodology developed in this study can also be applied to other organs, e.g., brain, spleen, kidney, pancreas and lungs. With further development for other organs using this technology, the end user list is virtually limitless. Organ phantoms that have established blood vessels with flow will make it possible for clinicians to practice a multitude of intravascular therapeutic procedures such as transarterial chemoembolization, transarterial mechanical embolization, selective intraarterial radiation therapy and transjugular intrahepatic portosystemic stent shunt, among others. In addition, realistic liver phantoms with lesions and anatomy-based vasculature would be useful tools to practice microcatheterization of vessels in the liver.

**Table 1:** Summary of the main features regarding the liver phantoms available on the market and developed for research purposes

	Multimodal	LUS	Anthropomorphic	Tumors (different types)	Blood vessels	Blood flow	Applications
Banovac et al. [7]	No (CT)	No	–	Yes (No)	No	No	Testing technologies for CT-guided needle targeting
Bao et al. [19]	Yes (US, CT)	Yes	No	No	No	No	US-to-CT registration
Chmarra et al. [3]	Yes (US, CT, MRI)	Yes	No	Yes (No)	Yes (portal vein)	No	Medical imaging development
Herline et al. [8]	No (CT)	No	Yes	Yes (No)	No	No	Surface-based registration
In et al. [14]	No (MRI)	No	No	Yes (No)	No	No	Calibration and training
Joe et al. [9]	No (CT)	No	–	No	No	No	Assessment of hepatic iron accumulation
Kao et al. [20]	Yes (PET/CT)	No	No	Yes (No)	No	No	Simulation of heterogeneous microsphere biodistribution of a postradioembolization liver
Murotani et al. [10]	No (CT)	No	No	Yes (No)	Yes	No	Analysis of the optimum CT reconstruction parameters
Olerud et al. [11]	No (CT)	No	Yes	Yes (Yes)	No	No	Assessment of image quality in CT and its relationship to radiation dose
Pacioni et al. [16]	No (US)	No	Yes	Yes (Yes)	Yes (portal vein and vena cava)	No	Training
Rube et al. [15]	No (MRI)	No	No	Yes (No)	Yes	No	Testing technology for MRI-guided needle targeting
Schindera et al. [12]	No (CT)	No	No	Yes (No)	No	No	Assessment of the impact of large patient size on the detection of hypovascular tumors
Schwaiger et al. [17]	No (US)	–	Yes	Yes (No)	Yes	No	Testing of US-based navigation technology
Shevchenko et al. [4]	Yes (US, CT)	Yes	Yes	Yes (No)	Yes	No	Testing of navigation technology and resection training
Sugimoto et al. [18]	No (US)	Yes	No	Yes (No)	No	No	Testing imaging systems for US-guided needle targeting
Widmann et al. [13]	No (CT)	No	Yes	Yes (No)	Yes	No	Testing imaging systems for CT-guided needle targeting
Anthropomorphic liver phantom (QRM, Moehrendorf, Germany)	No (CT)	No	Yes	Yes (Yes)	No	No	Medical imaging development and training
FAST exam real-time ultrasound training model (CAE Healthcare, CAE Healthcare, Sarasota, FL, USA)	No (US)	No	Yes	No	No	Yes (internal bleeding)	Training
IOUSFAN (Kyoto Kagaku Co., Ltd, Kyoto, Japan) [6]	No (US)	Yes	Yes	Yes (Yes)	Yes (portal vein, hepatic vein and aorta)	No	Training
Triple Modality 3D Abdominal Phantom (CIRS, Norfolk, VA, USA) [5]	Yes (US, CT, MRI)	No	Yes	Yes (No)	Yes (portal vein and aorta)	No	Medical imaging development and biopsy training

*LUS* Laparoscopic ultrasound

## Conclusion

We have established a method for producing a lasting artificial liver phantom based on real organ anatomy, including flow functionality. We were also able to demonstrate multimodal imaging capabilities of the phantom. The phantom is equally suitable for practicing simple diagnostic procedures such as bed-side ultrasound to high-end minimally invasive surgical navigation procedures requiring several imaging modalities simultaneously. The phantom was designed for clinical training as well as technological system development purposes. It can replace live animal experimentation and is superior to computer simulations for both simple clinical diagnostics and high-end surgical procedures limited to a single organ.

## References

[1] Rethy A, Langø T, Mårvik R (2013). Laparoscopic ultrasound for hepatocellular carcinoma and colorectal liver metastasis: an overview. Surg Laparosc Endosc Percutaneous Tech.

[2] Langø T, Vijayan S, Rethy A, Våpenstad C, Solberg OV, Mårvik R, Johnsen G, Hernes TN (2012). Navigated laparoscopic ultrasound in abdominal soft tissue surgery: technological overview and perspectives. Int J Comput Assist Radiol Surg.

[3] Chmarra MK, Hansen R, Mårvik R, Langø T (2013). Multimodal phantom of liver tissue. PLoS ONE.

[4] Shevchenko N, Schwaiger J, Markert M, Flatz W, Lueth TC (2011). Evaluation of a resectable ultrasound liver phantom for testing of surgical navigation systems. Conf Proc IEEE Eng Med Biol Soc.

[5] Computerized Imaging Reference Systems, Inc. (2017) CIRS, Triple modality 3D abdominal phantom, Model 057A. http://www.cirsinc.com/products/modality/65/triple-modality-3d-abdominal-phantom/. Accessed 8 May 2017

[6] Kyoto Kagaku Co., Ltd. (2017) IOUSFAN, Abdominal intraoperative & laparoscopic ultrasound phantom. 2017. http://www.kyotokagaku.com/products/detail03/us-3.html. Accessed 8 May 2017

[7] Banovac F, Tang J, Xu S, Lindisch D, Chung HY, Levy EB, Chang T, McCullough MF, Yaniv Z, Wood BJ, Cleary K (2005). Precision targeting of liver lesions using a novel electromagnetic navigation device in physiologic phantom and swine. Med Phys.

[8] Herline AJ, Herring JL, Stefansic JD, Chapman WC, Galloway RL, Dawant BM (2000). Surface registration for use in interactive, image-guided liver surgery. Comput Aided Surg.

[9] Joe E, Kim SH, Lee KB, Jang J-J, Lee JY, Lee JM, Han JK, Choi BI (2012). Feasibility and accuracy of dual-source dual-energy CT for noninvasive determination of hepatic iron accumulation. Radiology.

[10] Murotani K, Kazuhiro M, Kawai N, Sato M, Minamiguchi H, Nakai M, Sonomura T, Hosokawa S, Nishioku T (2013). Optimum CT reconstruction parameters for vascular and hepatocellular carcinoma models in a liver phantom with multi-level dynamic computed tomography with 64 detector rows: a basic study. Radiol Phys Technol.

[11] Olerud HM, Olsen JB, Skretting A (1999). An anthropomorphic phantom for receiver operating characteristic studies in CT imaging of liver lesions. Br J Radiol.

[12] Schindera ST, Torrente JC, Ruder TD, Hoppe H, Marin D, Nelson RC, Szucs-Farkas Z (2011). Decreased detection of hypovascular liver tumors with MDCT in obese patients: a phantom study. Am J Roentgenol.

[13] Widmann G, Wallach D, Toporek G, Schullian P, Weber S, Bale R (2013). Angiographic C-arm CT- versus MDCT-guided stereotactic punctures of liver lesions: nonrigid phantom study. Am J Roentgenol.

[14] In E, Naguib H, Haider M (2014). Mechanical stability analysis of carrageenan-based polymer gel for magnetic resonance imaging liver phantom with lesion particles. J Med Imaging (Bellingham).

[15] Rube MA, Holbrook AB, Cox BF, Buciuc R, Melzer A (2015). Wireless mobile technology to improve workflow and feasibility of MR-guided percutaneous interventions. Int J Comput Assist Radiol Surg.

[16] Pacioni A, Carbone M, Freschi C, Viglialoro R, Ferrari V, Ferrari M (2015). Patient-specific ultrasound liver phantom: materials and fabrication method. Int J Comput Assist Radiol Surg.

[17] Schwaiger J, Markert M, Shevchenko N, Lueth TC (2011). The effects of real-time image navigation in operative liver surgery. Int J Comput Assist Radiol Surg.

[18] Sugimoto K, Moriyasu F, Shiraishi J, Yamada M, Imai Y (2011). A phantom study comparing ultrasound-guided liver tumor puncture using new real-time 3D ultrasound and conventional 2D ultrasound. Am J Roentgenol.

[19] Bao P, Warmath J, Galloway R, Herline A (2005). Ultrasound-to-computer-tomography registration for image-guided laparoscopic liver surgery. Surg Endosc.

[20] Kao YH, Luddington OS, Culleton SR, Francis RJ, Boucek JA (2014). A gelatin liver phantom of suspended 90Y resin microspheres to simulate the physiologic microsphere biodistribution of a postradioembolization liver. J Nucl Med Technol.

[21] Culjat MO, Goldenberg D, Tewari P, Singh RS (2010). A review of tissue substitutes for ultrasound imaging. Ultrasound Med Biol.

[22] Kato H, Kuroda M, Yoshimura K, Yoshida A, Hanamoto K, Kawasaki S, Shibuya K, Kanazawa S (2005). Composition of MRI phantom equivalent to human tissues. Med Phys.

[23] Pogue BW, Patterson MS (2006). Review of tissue simulating phantoms for optical spectroscopy, imaging and dosimetry. J Biomed Opt.

[24] Kondo T, Kitatuji M, Kanda H (2005). New tissue mimicking materials for ultrasound phantoms. Ultrason Symp IEEE.

[25] Fromageau J, Brusseau E, Vray D, Gimenez G, Delachartre P (2003). Characterization of PVA cryogel for intravascular ultrasound elasticity imaging?. IEEE Trans Ultrason Ferroelectr.

[26] Surry KJ, Austin HJ, Fenster A, Peters TM (2004) Poly(vinyl alcohol) cryogel phantoms for use in ultrasound and MR imaging. Phys Med Biol 49:5529–5546 10.1088/0031-9155/49/24/009 15724540

[27] Mazzara GP, Briggs RW, Wu Z, Steinbach BG (1996). Use of a modified polysaccharide gel in developing breast phantom for MRI. Magn Reson Imaging.

[28] Zell K, Sperl JI, Vogel MW, Niessner R, Haisch C (2007). Acoustical properties of selected tissue phantom materials for ultrasound imaging. Phys Med Biol.

[29] Bays R, Wagnières G, Robert D, Theumann JF, Vitkin A, Savary JF, Monnier P, van den Bergh H (1997). Three-dimensional optical phantom and its application in photodynamic therapy. Lasers Surg Med.

[30] Waterworth MD, Tarte BJ, Joblin AJ, van Doorn T, Niesler HE (1995). Optical transmission properties of homogenised milk used as a phantom material in visible wavelength imaging. Aust Phys Eng Sci Med.

[31] Mitic G, Kölzer J, Otto J, Plies E, Sölkner G, Zinth W (1994). Time-gated transillumination of biological tissues and tissue-like phantoms. Appl Opt.

[32] Inford J, Shalev S, Bews J, Brown R, Schipper H (1986). Development of a tissue-equivalent phantom for diaphanography. Med Phys.

[33] Moes CJM, van Gemert MJ, Star WM, Marijnissen JPA, Prahl SA (1989). Measurements and calculations of the energy fluence rate in a scattering and absorbing phantom at 633 nm. Appl Opt.

[34] van Staveren HJ, Moes CJM, van Marle J, Prahl SA, van Gemert MJC (1991). Light scattering in intralipid-10% in the wave-length range of 400–1100 nm. Appl Opt.

[35] Flock ST, Jacques SL, Wilson BC, Star WM, Vangemert MJC (1992). Optical properties of Intralipid: a phantom medium for light propagation studies. Lasers Surg Med.

[36] Krucker T, Lang A, Meyer EP (2006). New polyurethane-based material for vascular corrosion casting with improved physical and imaging characteristics. Microsc Res Tech.

[37] Verli FD, Rossi-Schneider TR, Schneider FL, Yurgel LS, de Souza MAL (2007). Vascular corrosion casting technique steps. Scanning.

[38] Askeland C, Solberg OV, Bakeng JBL, Reinertsen I, Tangen GA, Hofstad EF, Iversen DH, Våpenstad C, Selbekk T, Langø T, Hernes TAN, Olav Leira H, Unsgård G, Lindseth F (2016). CustusX: an open-source research platform for image-guided therapy. Int J Comput Assist Radiol Surg.

[39] Bø LE, Hofstad EF, Lindseth F, Hernes TA (2015). Versatile robotic probe calibration for position tracking in ultrasound imaging. Phys Med Biol.

[40] O’Brien EK, Wayne DB, Barsness KA, McGaghie WC, Barsuk JH (2016). Use of 3D printing for medical education models in transplantation medicine: a critical review. Curr Transplant Rep.

[41] Kusaka M, Sugimoto M, Fukami M, Sasaki H, Takenaka M, Anraku T, Ito T, Kenmochi T, Shiroki R, Hoshinaga K (2015). Initial experience with a tailor-made simulation and navigation program using a 3-D printer model of kidney transplantation surgery. Transplant Proc.

